# Modulating ATP binding cassette transporters in papillary renal cell carcinoma type 2 enhances its response to targeted molecular therapy

**DOI:** 10.1002/1878-0261.12346

**Published:** 2018-08-23

**Authors:** Rola M. Saleeb, Mina Farag, Zsuzsanna Lichner, Fadi Brimo, Jenni Bartlett, Georg Bjarnason, Antonio Finelli, Fabio Rontondo, Michelle R. Downes, George M. Yousef

**Affiliations:** ^1^ Department of Laboratory Medicine, and the Keenan Research Centre for Biomedical Science at the Li Ka Shing Knowledge Institute St. Michael's Hospital Toronto Canada; ^2^ Department of Laboratory Medicine and Pathobiology University of Toronto Canada; ^3^ Department of Pathology McGill University Health Center Montreal Canada; ^4^ Division of Medical Oncology and Hematology Sunnybrook Health Sciences Toronto Canada; ^5^ Division of Urology Department of Surgery University Health Network Toronto Canada; ^6^ Department of Pathology Sunnybrook Health Sciences Centre Toronto Canada

**Keywords:** ABC transporters, papillary renal cell carcinoma subtypes, papillary renal cell carcinoma type 2, renal cell carcinoma cell lines, targeted therapy

## Abstract

Papillary renal cell carcinoma (PRCC) is the most common nonclear cell RCCs and is known to comprise two histological subtypes. PRCC2 is more aggressive and is molecularly distinct from the other subtypes. Despite this, PRCCs are treated together as one entity, and they show poor response to the current therapies that do not target pathways implicated in their pathogenesis. We have previously detected ABCC2 (an ABC transporter), VEGF, and mTOR pathways to be enriched in PRCC2. In this study, we assess the therapeutic potential of targeting these pathways in PRCC2. Twenty RCC cell lines from the Cancer Cell Encyclopedia were compared to the Cancer Genome Atlas PRCC cohort (290), to identify representative PRCC2 cell lines. Cell lines were further validated in xenograft models. Selected cell lines were treated *in vitro* and *in vivo* (mice models) under five different conditions, untreated, anti‐VEGF (sunitinib), ABCC2 blocker (MK571), mTOR inhibitor (everolimus) and sunitinib + MK571. Sunitinib +ABCC2 blocker group showed a significant response to therapy compared to the other treatment groups both *in vitro* (*P ≤ *0.0001) and *in vivo* (*P* = 0.0132). ABCC2 blockage resulted in higher sunitinib uptake, both *in vitro* (*P *=* *0.0016) and *in vivo* (*P *=* *0.0031). Everolimus group demonstrated the second best response *in vivo*. The double‐treatment group showed the highest apoptotic rate and lowest proliferation rate. There is an urgent need for individualized therapies of RCC subtypes that take into account their specific biology. Our results demonstrate that combined targeted therapy with sunitinib and ABCC2 blocker in PRCC2 has therapeutic potential. The results are likewise potentially significant for other ABCC2 high tumors. However, the results are preliminary and clinical trials are needed to confirm these effects in PRCC2 patients.

**Abbreviations**
ABC transporters: ATP binding cassette
ABCC2: ABC binding cassette subfamily C member 2
CCRCC: clear cell renal cell carcinoma
Chr: chromosome
PRCC: papillary renal cell carcinoma

## Introduction

Renal cell carcinomas (RCCs) are tumors that arise from renal tubules. They represent ~90% of adult kidney tumors and one of the most prevalent malignancies worldwide (Afriansyah *et al*., 2016; Kumar and Kapoor, 2017). Due to the lack of predictable early signs and symptoms of the disease, 30% of the cases are discovered at a locally advanced or metastatic stage(Kumar and Kapoor, 2017). At that point, the 5‐year survival rate becomes extremely poor (10–50%) (Afriansyah *et al*., 2016; Kumar and Kapoor, 2017).

RCCs are, however, composed of multiple histological types, the most common of which are the clear cell RCCs (CCRCC) (~75%), and the second most common are papillary RCC (PRCC) (10–15%) which is comprised of multiple subtypes (Saleeb *et al*., 2016). We and others have shown that PRCC subtypes are also molecularly and prognostically distinct (Saleeb *et al*., 2016; TCGA, 2016; Yang *et al*., 2005). Other RCC subtypes as chromophobe, MiT family translocation RCCs and other rare types collectively constitute the remaining 5–10% (Moch *et al*., 2016).

Targeted molecular therapies are currently the standard of care for metastatic CCRCCs. These include first‐line therapies vascular endothelial growth factor (VEGF) tyrosine kinase inhibitors (TKIs), for example, sunitinib and pazopanib, and second‐line therapies the mammalian target of rapamycin (mTOR) inhibitors (everolimus and temsirolimus), and the recently approved PD‐1 immune checkpoint inhibitor nivolumab (Ciccarese *et al*., 2015, 2017; Kumar and Kapoor, 2017). There is, however, a substantial lack of evidence regarding the management of nonclear cell RCC (Giles *et al*., 2017). While they are treated empirically as CCRCCs, they do not respond as well as the CCRCC counterpart.

In our previous studies, we have confirmed the distinct molecular profiles of PRCC subtypes 1 and 2. We also uncovered specific molecular pathways and biomarkers that can influence PRCC subtypes response to therapy (Saleeb *et al*., 2016, 2017). PRCC1, as noted from previous literature, had much better prognosis, compared to PRCC2 (Klatte *et al*., 2010; Pignot *et al*., 2007). Pignot *et al*. found the PRCC1 tumors to have significantly better overall survival and disease‐free survival than PRCC2. Their PRCC1 tumors also had significantly lower TNM stage diseases, and the differences in survival retained significance on multivariate analysis (Pignot *et al*., 2007). In our previous work, gene set enrichment analysis indicated that PRCC1 had enrichment in the MET, WNT, and NOTCH pathways, while in comparison, PRCC2 had enrichment in the mTOR, VEGF, and NRF2‐ARE pathways (Saleeb *et al*., 2016). These findings were consistent with the findings of the Cancer Genome Atlas (TCGA) and other reports (Ooi *et al*., 2013; TCGA, 2016). The mTOR and VEGF pathways are known to activate each other in a positive feedback loop manner (Guo *et al*., 2015). ABCC2 and other drug transporters are activated downstream to the ARE pathway (Jeong *et al*., 2015; Taguchi *et al*., 2011; TCGA, 2016). Interestingly, HIF1α in stabilized and increased in response to ARE activation and in turn HIF1α is known to induce VEGF (Taguchi *et al*., 2011).

Additionally, PRCC2 highly expressed the ABC drug transporter ABCC2 at the transcriptomic and proteomic level (79 times higher in PRCC2 than PRCC1), with high statistical significance (Saleeb *et al*., 2016, 2017). ABCC2 is known to be activated downstream to the NRF2‐ARE pathway which is specific to the PRCC2 biology (Jeong *et al*., 2015; TCGA, 2016). We have also shown that ABCC2 can be used as a prognostic marker to classify the PRCC tumors. ABCC2 belongs to the family of ABC transporters and is reported to contribute to chemotherapy resistance and thus called multidrug‐resistant protein 2 (MRP2) (Jeong *et al*., 2015). Reports suggest that ABCC2 and other ABC transporters induce cancer resistance to TKIs (Kathawala *et al*., 2015; Shibayama *et al*., 2011). Hence, it might contribute to the observed developed resistance in PRCC2 by actively transporting the drugs out of the tumor cells.

Therapeutic clinical trials for nonclear cell RCC are extremely limited, with small patient cohorts, and mostly all yielding disappointing results (Ciccarese *et al*., 2017; Courthod *et al*., 2015; Giles *et al*., 2017). One major hindrance to these trials is the inclusion of all nonclear cell RCCs as one category, despite the fact that they are known to be phenotypically and molecularly diverse (Armstrong *et al*., 2016; Giles *et al*., 2017; Ravaud *et al*., 2015; Tannir *et al*., 2016). There is considerable need for therapies and clinical trials that take into account the oncogenic pathways specific to each of these RCC subtypes (Giles *et al*., 2017; Kumar and Kapoor, 2017).

There are only two trials that have attempted to assess differences in response to therapy between the two subtypes of PRCC; however, small patient cohorts limited both trials (Escudier *et al*., 2016; Ravaud *et al*., 2015). There are currently clinical trials assessing the value of targeting the MET pathway in PRCC (Giles *et al*., 2017). MET pathway is significantly enriched in PRCC1 (Saleeb *et al*., 2016; TCGA, 2016); however, there are no trials focusing on the more aggressive PRCC2 phenotype.

We hypothesize that PRCC2 could benefit from targeting pathways that are specifically enriched in its phenotype. Blockage of ABCC2 in addition to the standard first‐line therapies of metastatic RCCs might be of added benefit to the tumor treatment. Also, tumors of pure PRCC2 biology would potentially benefit from mTOR inhibitors in contrast to other treatment modalities. In this study, we proceed to assess the value of targeting these pathways both *in vitro* and *in vivo* in PRCC2.

## Methods

### Ethics approval, Public genomic, and clinical data extraction

Ethics approval was obtained through our institution's ethics board. The local animal care committee approved all animal experiments. Publically available databases used were the TCGA, GEO (Gene expression Omnibus), and CCLE (Cancer Cell Line Encyclopedia‐Broad Institute). Gene expressions (mRNA seq, RSEM values) were downloaded for the KIRP 290 PRCC cohort from the TCGA Web site (our published study) (Saleeb *et al*., 2016). Gene expression (microarray) data for 20 Renal Cancer cell lines from the CCLE was explored and extracted through GEO (786‐O, KMRC‐3, KMRC‐2, KMRC‐20, KMRC‐1, CAL‐54, Caki‐1, Caki‐2, BFTC‐909, ACHN, A‐704, A‐498, 769‐P, VMRC‐RCZ, VMRC‐RCW, TUHR4TKB, TUHR14TKB, TUHR10TKB, SNU‐1272, RCC10RGB) (Barretina *et al*., 2012).

### Selection of cell lines

First, we identified CAL‐54 (RCC cell line) as a PRCC1 cell line based on literature evidence of its papillary nature (Gioanni *et al*., 1996; Sinha *et al*., 2017), low ABCC2 expression, and its known chromosomal aberrations (gains in Chr 7 & 17). To further validate the morphology and immune phenotypes of CAL‐54 as a PRCC1 representative cell line, the cells were cultured *in vitro*, after which 10^6^ cells were injected subcutaneously in immunocompromised outbred athymic nude (homozygous Foxn1 ^nu^) mice (4 mice). Tumors were harvested and sections were histologically examined for cell lines morphology and IHC profiles and compared to those of PRCC1. Similar to PRCC1, the histology of the mice tumors showed a tubulo‐papillary architecture of small low‐grade tumor cells, linear nuclear arrangement, and negative staining for ABCC2 IHC (Fig. S1). Next, the CAL‐54 gene expression signature (from CCLE) was used as a comparison against which we elicited the top expressed genes in the other available 19 RCC CCLE cell lines. That comparison was correlated with the TCGA tumors comparison (PRCC1 vs PRCC2) to elicit best‐fit cell line to PRCC2.

### Cell culture *in vitro*

The CAKI‐2 and CAL‐54 RCC cells were obtained from the American Type Culture Collection (ATCC, Manassas, VA, USA) and the Deutsche Sammlung von Mikroorganismen und Zellkulturen (DSMZ, Germany), respectively. The former was grown in McCoy cell media with 10% fetal bovine serum (FBS), while the latter was grown in DMEM cell media with 15% FBS, 0.4 μg·mL^−1^ hydrocortisone, and 10 ng·mL^−1^ EGF.

### *In vitro* treatment

Cells were plated at 1.0 × 10^3^ cells per well in a 96‐well plate and after 24 h grouped into the five treatment conditions, untreated, treated sunitinib 1.0 μm, treated with MK‐571 25 μm (ABCC2 blocker) (Tang *et al*., 2002), treated with combined MK‐571 + sunitinib, and treated with everolimus 50 nM (Lane *et al*., 2009). Cellular viability was measured using the WST‐1 cell proliferation colorimetric assay (Roche Applied Science, Indianapolis, IN, USA) at day 2, 3, and 9 post‐treatment. Described resistance index equation was used to measure the cytotoxic effect of the medications on the cells(Pénzváltó *et al*., 2013). The exact equation is:

RI=(N2−Npre)/(Npost−Npre)

Npre is the medium absorbance value of precontrol (representing the number of cells at the beginning of the treatment), Npost is the medium absorbance value of control (representing the number of cells with no treatment at the end of the treatment period), and N2 is the medium absorbance value of treated cells at the end of the treatment period.

### Effect of blocking ABCC2 on sunitinib and other drug uptake *in vitro* and *in vivo*

The spectral properties of sunitinib were noted from the previous literature (Nowak‐Sliwinska *et al*., 2015). Brilliant violet 510 (BV510) fluorochrome was identified as having very similar light absorbance, excitation, and emission properties as sunitinib. The BV510 spectral range of detection was used to detect sunitinib fluorescence intensity in treated cells. Cell lines were cultured and treated with sunitinib ± MK571. After 4 days of treatment, cells were trypsinized and washed with PBS‐1% and assessed for sunitinib presence with flow cytometry. All experiments were repeated in triplicates.

Similarly, CAKI‐2 cell line tumors grown in mice were harvested after 8 weeks of treatment. Tumors were dissociated through mincing tumor sample and then incubated for 30 min at 37 °C with hyaluronidase and collagenase enzyme mix. Tumor cells were further passed through a 70‐micron filter mesh, and then, cells were washed with PBS for subsequent analysis with flow cytometry.

CAKI‐2 cells seeded in 96‐well plates and treated with MK‐571 were stained with Hoechst 33342 (DNA dye). The plate was scanned with image express analyzer to detect the number of cells with dye uptake. Subsequently, the cells were fixed with paraformaldehyde (PFA), subjected to the Hoechst dye again, and rescanned with the image express.

### *In vivo* validation

Mice bought from ‘The Jackson laboratory’ were the immunocompromised strains outbred athymic nude (homozygous Foxn1 ^nu^) mice and NOD SCID (severe combined immunodeficiency) gamma mice.

CAKI‐2 cells were grown to 80% confluency and trypsinized, into single‐cell suspension. 10^6^ CAKI‐2 cells were suspended in a 100ul of saline and added to another 100ul of Matrigel and then injected subcutaneously above the mice flanks Treatment started when the tumors reached 100 mm^3^ using the formula: length x (diameter)^2^ x π /6, where length is the longest dimension and diameter is the shortest dimension (Zhu *et al*., 2012). Mice were divided into five treatment groups with an average of 4–6 mice per arm: untreated (control), treated with MK571 only (25 mg·kg^−1^), sunitinib only (50 mg·kg^−1^), everolimus only (2.5 mg·kg^−1^), sunitinib + MK571, and everolimus + MK571(Hara *et al*., 2011; Karam *et al*., 2011; Lane *et al*., 2009; Zhu *et al*., 2012). The treatment was given through oral gavage 5 days on and 2 days off for the period of 8 weeks. The response was assessed through tumor growth curves and through evaluation of metastasis at the experiment end point (mice autopsy). Percentage of apoptotic cells was assessed at mice end point after tumor dissociation, with the Annexin V flow cytometry apoptosis assay. The proliferation of tumor cells was assessed with the Ki67 immunohistochemical stain on mice tumors. Ki67 staining was quantified using the aperio image analysis software (Leica Biosystems Group of Companies, Wetzlar, Germany).

### Immunohistochemistry

Immunohistochemistry was performed using Ki67 and ABCC2 antibodies. The Ki67 was performed using a Ventana automated system. Ki67 is a marker of cell proliferation (detected by nuclear staining), as it is present in all active phases of the cell cycle (de Sousa e Melo *et al*., 2017). IHC staining of ABCC2 was achieved by the streptavidin–biotin–peroxidase complex protocol using an ABCC2‐specific mouse monoclonal antibody (Monosan, UDEN, the Netherlands; Cat# MON9026; dilution 1 : 200). FFPE normal kidney tissue served as a positive control, while substitution of the primary antibodies with PBS served as a negative control.

### Quantitative image analysis and statistical analysis

Mice tumor slides stained with Ki67 were scanned and analyzed with the aperio image analysis software. The algorithm combines staining intensity and percentage positivity to provide a combined score (Kolin *et al*., 2014). Statistical analysis was performed using the GraphPad 7 prism and spss statistical software packages (Chicago, IL, USA).

## Results

### Identification of cell lines with best molecular, morphological, and immunophenotypical correlation with PRCC2

From the PRCC TCGA cohort, a distinct PRCC2 mRNA signature of 500 genes (compared to PRCC1) was elicited (Saleeb *et al*., 2016) and compared with the top 500 genes for each of the CCLE cell lines (compared to CAL‐54) using Pearson correlation coefficient r and linear regression r2, as previously described (Malone and Oliver, 2011). As the ARE pathway is known to be enriched in PRCC2, an ARE PRCC2 gene signature (ABCC2, ACTA2, ACTC1, ACTG2, EPHX1, FTL, GCLM, GPX2, GSR, GSTA1, GSTA2, NQO1, PRKCE, SQSTM1, TXNRD1, AKR1B10, AKR1C1, AKR1C3, SRXN1) (Ooi *et al*., 2013; TCGA, 2016) was used to assess the correlation between the PRCC2 and each of the RCC cell lines. The bioinformatic statistical analysis was performed using graphpad prism 7 and spss statistical software (San Diego, CA, USA).

**Figure 1:**
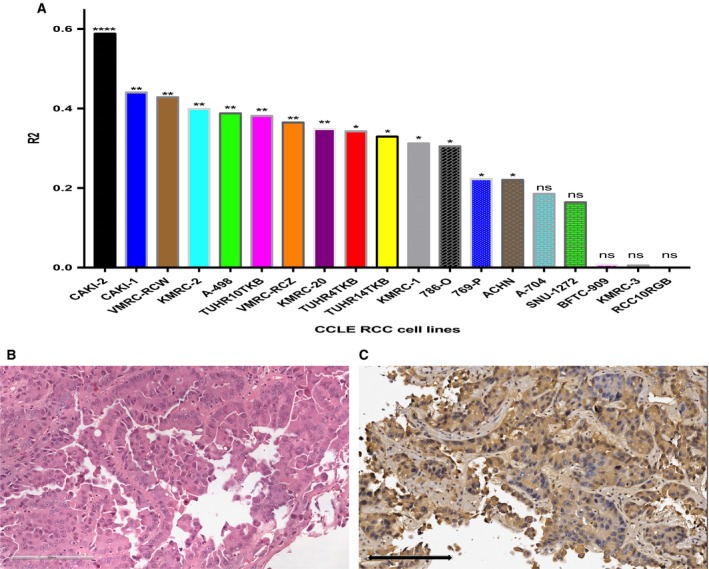
The CAKI‐2 cell line represents PRCC2. (A) Comprehensive bioinformatic analysis of the 19 renal cell carcinoma cell lines from the Cancer Cell Line Encyclopedia (CCLE) revealed CAKI‐2 as best match to PRCC2. Coefficient of determination statistical test was used to compare the expression of ARE pathway genes between the cell lines and PRCC2 (nonsignificant: ns, *P *>* *0.05:*, *P ≤ *0.05:**, *P *≤* *0.01:***, P ≤* *0.001:****, *P *≤* *0.0001). (B) Histological sections of CAKI‐2 cells xenografted in mouse model showed typical PRCC2 morphology formed of papillary growth with large cells, abundant cytoplasm and prominent nucleoli, and pseudostratification. (C) The cells stained strong diffuse positive for ABCC2, in agreement with the expression level of this protein in PRCC2 tumors. Scale bar = 200 μm.

Of the 19 RCC cell lines, CAKI‐2 showed excellent correlation with PRCC2 top matched genes (Pearson correlation coefficient r: 0.79, r^2^:0.63 (*P* ≤ 0.0001), as well as best correlation with the ARE pathway genes (Pearson correlation coefficient r:0.77, r^2^:0.59 (*P* = 0.0002) Figs 1A and  S2.

To further validate the morphology and immune phenotypes of our selected cell lines, CAKI‐2 was cultured *in vitro*, after which 10^6^ cells were injected subcutaneously in immunocompromised outbred athymic nude (homozygous Foxn1 ^nu^) mice (6 mice). Tumors were harvested and sections were histologically examined for cell lines morphology and IHC profiles and compared to those of PRCC2. All of the six injected nude mice developed tumors with papillary morphology and had large eosinophilic cells, with nuclear pseudostratification and prominent nucleoli, consistent with what is known as a PRCC2 morphology (Fig. 1B). Additionally, the tumors demonstrated diffuse strong staining with ABCC2 (Fig. 1C) as we have previously described in the PRCC2 subtype (Saleeb *et al*., 2017). Taken together, the morphology, immunostaining pattern, and molecular analyses show that CAKI‐2 can serve as a representative model for PRCC2. This is consistent with Brodaczewska *et al*. recent review on RCC cell lines describing multiple evidence linking CAKI‐2 to PRCC (Brodaczewska *et al*., 2016). Additionally, CAKI‐2 harbors chromosome 8 aberrations, which has also been described in PRCC2 (Furge *et al*., 2007; Saleeb *et al*., 2017).

### *In vitro* treatment of PRCC2 cell line shows the best response achieved with double‐treatment sunitinib and ABCC2 blocker therapy

**Figure 2:**
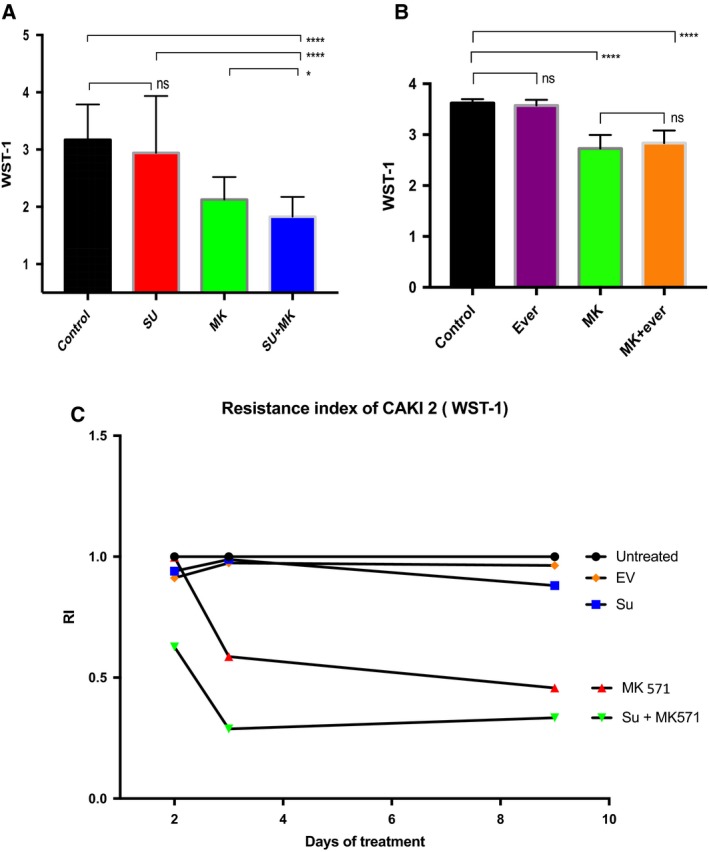
*In vitro* response of CAKI‐2 (PRCC2 cell line) to different treatment modalities, late (day 9) responses are shown. (A) The addition of ABCC2 blocker to sunitinib results in significant decrease in viable cell count. (control *n* = 19, sunitinib *n* = 17, MK *n* = 20, sunitinib + MK571 *n* = 20) (B) Similar analysis adding MK571 to everolimus did not produce significant decrease in viable cell count than MK571 alone (control *n* = 21, everolimus *n* = 23, MK *n* = 23, everolimus + MK571, *n* = 23). Two‐tailed *t*‐test used for the analysis of A and B, error bars represent SD. (Nonsignificant: ns, *P *>* *0.05:*, *P ≤ *0.05:**, *P *≤* *0.01:***, P ≤* *0.001:****, *P *≤* *0.0001.) (C) Response to treatment as assessed by resistance index (RI) to the different treatments. RI is calculated through a special formula that compares the cells response to the control group, the control group is given the RI = 1, and a more drug‐sensitive response = <1.

We first assessed CAKI‐2 cell susceptibility to sunitinib (current first‐line metastatic RCC treatment) ± MK571. The group of cells that were exposed to the dual treatment with both sunitinib and blocker was the most sensitive to treatment (Fig. 2A), using both the WST‐1 viability assay and the calculated resistant index. The results indicate that ABCC2 as a drug transporter might play a role in the sunitinib medication influence of the treated cancer cells (Warta *et al*., 2014; Zhang *et al*., 2014). The group of cells treated with MK571 only also showed a considerable reduction in cell viability. This is in agreement with recent reports that ABC transporters are directly implicated in the biology of the tumors rather than being merely drug transport pumps(Henderson *et al*., 2011; Wu *et al*., 2017).

We performed a similar *in vitro* experiment assessing the effect of everolimus with and without MK571 on the cancer cells. Interestingly, the combination of blocker (MK571) and everolimus did not produce significant differences in cell viability versus the blocker alone (Fig. 2B).

Assessing response using resistance index again showed a superior response to both MK571 ±  sunitinib (Fig. 2C).

### *In vitro*, ABCC2 induces active export of medications

**Figure 3:**
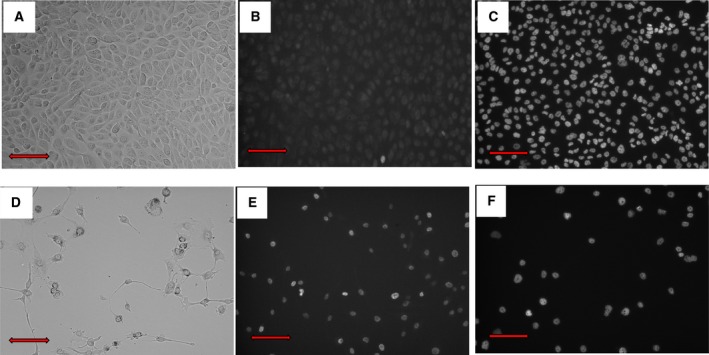
*In vitro* assessment of the drug transporter pump effect (CAKI‐2 PRCC2 cell line.) (A) Untreated cells, (B) Untreated cells stained with Hoescht permeable stain showed no staining indicating active transport of the dye outside the cell, (C) after blocking the receptor, there is a significant increase in signal indicating dye retention. (D**)** MK571 (ABCC2 blocker)‐treated cells. Treated cells stained for the dye before (E) and after fixation (F). Red scale bar = 200 μm.

To validate the activity of ABCC2 drug transporter pump in PRCC2, CAKI‐2 cells were grown in 96‐well plates. At the end of 9 days, untreated and MK571 (ABCC2 blocker) treated groups were stained with the cell permeable Hoescht 33342 DNA dye and then subjected to image screening and visualization using the Xpress image analyzer. MK571‐treated cells showed considerable dye uptake (indicating dye retention), whereas untreated cells failed to stain indicating an active export of the Hoescht dye by ABCC2. Then, we proceeded to fix the cells with paraformaldehyde (PFA) to terminate the ABCC2 drug transporter pump effect. Fixed untreated cells showed a significant increase in staining after blocking the ABCC2 effect with fixation, *P* = 1.33275E‐15. While previously blocker treated cells did not show a significant change in stained cells after fixation, *P* = 0.73 (Fig. 3). The results indicate that the lack of staining in blocker untreated viable cells was an active process as in an ATP transporter pump, while adding the blocker to viable cells resulted in dye uptake without the need for fixation.

**Figure 4:**
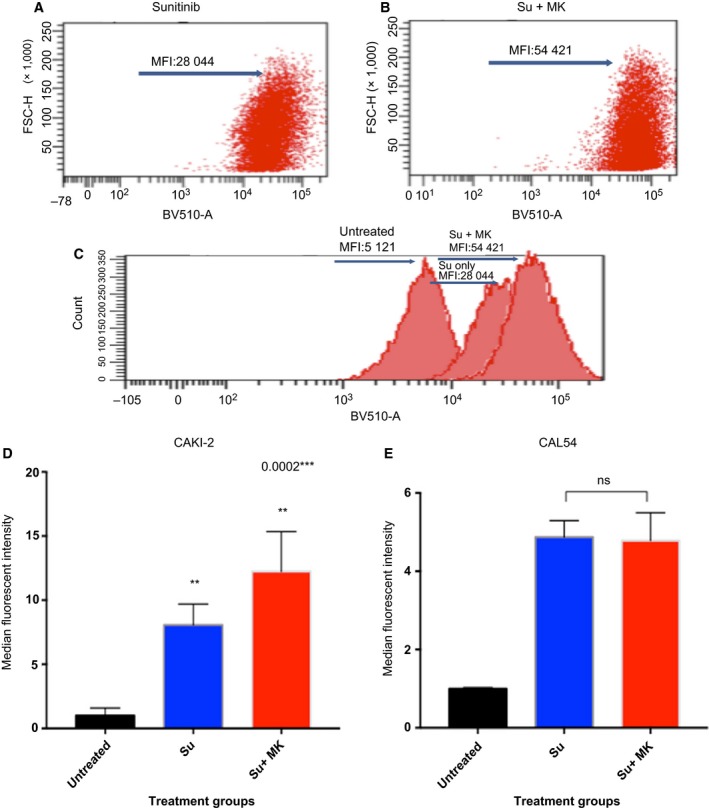
Sunitinib uptake of PRCC2 CAKI‐2 cells is increased with blockage of ABCC2. (A) sunitinib (Su) only treated cells show Su median fluorescence intensity (MFI) of 28 044. (B) Adding MK571 (ABCC2 blocker) increases Su MFI to 54 421. (C) Histogram of the MFI shift. (D) A bar graph representation of the data presented in A–C in CAKI‐2 cell line. (All groups *n* = 3). One‐way ANOVA statistical test, error bars represent SD (E). CAL54 RCC cell line (PRCC1 equivalent and low ABCC2 expression) shows no significant increase in sunitinib uptake by flow cytometry. (All groups *n* = 3) Two‐tailed t‐test used, error bars represent SD (nonsignificant: ns, *P *>* *0.05:*, *P ≤ *0.05:**, *P *≤* *0.01:***, P ≤* *0.001:****, *P *≤* *0.0001).

To further confirm that ABCC2 contributes to drug resistance through pumping out medications, we measured sunitinib uptake in CAKI‐2 cells through the detection of its known spectral range in flow cytometry (Nowak‐Sliwinska *et al*., 2015). The results show a near doubling of the uptake of sunitinib after the addition of MK571, from 28 044 median fluorescence intensity (MFI) to 54 421 MFI (Fig. 4A–D). To further validate our findings, we assessed sunitinib uptake ± ABCC2 blockage in the CAL54 cell lines (represent PRCC1 with low ABCC2 expression). Sunitinib uptake was not increased upon addition of MK571 (Fig. 4E).

### *In vivo* validation, tumor mouse models exhibit the highest response to therapy in the sunitinib + ABCC2 blocker group

**Figure 5:**
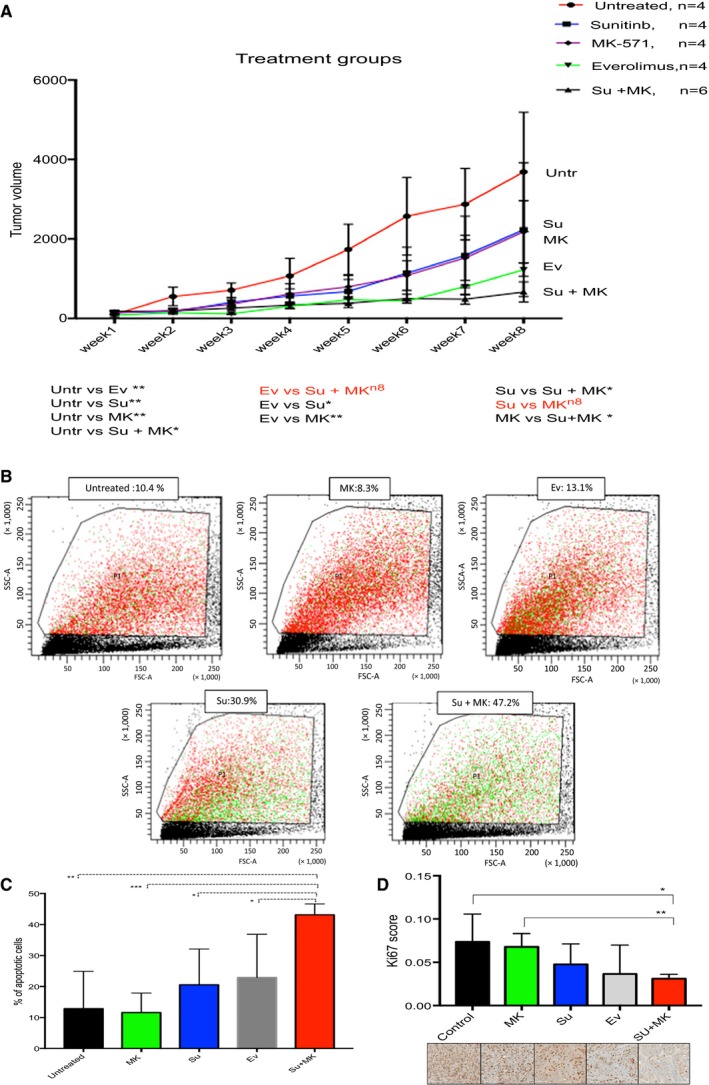
*In vivo* validation of treatment effect, CAKI‐2 cell line grown in mice. (A) Tumor growth curves in mouse models subjected to five treatment conditions; untreated, sunitinib, MK‐571, everolimus and sunitinib + MK571. Best response is shown in the double‐treatment and everolimus groups. (Untreated *n* = 4, sunitinib = 4, MK‐571 = 4, everolimus *n* = 4, sunitinib + MK571 = 6), error bars represent SEM. Paired *t*‐test is used for the analysis (B) Annexin V apoptosis assay assessing the percentage of apoptotic cells in the different treatment conditions. Highest level of apoptosis was found in the double‐treatment (sunitinib + MK571) group. Apoptotic cells stain green. (C) A bar graph showing the double‐treatment group (sunitinib + MK571) to cause a significant increase in apoptotic cells in comparison with the other treatment groups (untreated *n* = 3, MK571 *n* = 4, sunitinib *n* = 3, everolimus *n* = 2, sunitinib + Mk571 *n* = 4). (D) Digital quantification of Ki67 IHC (proliferation marker) of mice tumors shows double‐treatment group to have significantly less proliferation than the other groups (control *n* = 2, MK571 *n* = 4, sunitinib *n* = 3, everolimus *n* = 3, sunitinib + MK 571 = 5). Two‐tailed t‐test used for the analysis of C and D, error bars represent standard deviation (nonsignificant: ns, *P *>* *0.05:*, *P ≤ *0.05:**, *P *≤* *0.01:***, P ≤* *0.001:****, *P *≤* *0.0001). Scale bar = 200 μm

Tumor mouse model was optimized in immunocompromised athymic nude and NOD SCID gamma mice. 10^6^ CAKI‐2 cells were injected subcutaneously. We used physiologically relevant doses of sunitinib, MK‐571, and everolimus. Mice were divided into five treatment groups: untreated, sunitinib, sunitinib + MK‐571, everolimus, and MK571 only (Armstrong *et al*., 2016; Hara *et al*., 2011; Karam *et al*., 2011; Lane *et al*., 2009; Zhu *et al*., 2012). Mice were treated for eight consecutive weeks. Similar to what was observed *in vitro*, the best tumor response (final tumor size and tumor rate of growth) was observed in the group that was exposed to the sunitinib and MK571 treatment (Fig. 5A). Average tumor volume after treatment for that group was 663 mm^3^ compared to 2230 mm^3^ in the sunitinib only treated group (*P* = 0.045) and 3686 mm^3^ in the control untreated group (*P* = 0.0132). The growth curves of everolimus‐treated group showed the second highest response to therapy, volume of 1225 mm^3^. Both everolimus and sunitinib + MK571 arms showed significant differences from the sunitinib treated arm. Sunitinib only and MK571 only treated groups showed similar responses to their corresponding therapy (2230 mm^3^ and 2176 mm^3^, respectively) with no significant differences between the two treatment arms.

To further confirm our results, the percentage of apoptotic cells among each treatment group was measured with the Annexin V assay by flow cytometry. The sunitinib + MK571 group showed the highest level of apoptosis (mean 43.125%), which was significantly higher (*P* = 0.0045) compared to other treatment groups (Fig. 5B,C). We further assessed proliferation in formalin‐fixed sections of tumor tissues, which were stained with the proliferative marker Ki67 and quantified with digital image analysis. Our findings revealed significant decrease in proliferation in the sunitinib + blocker group (*P* = 0.0197) (Fig. 5D).

Lastly, we histologically examined the mice tissue organs for evidence of response, progression, and metastasis in the five treatment groups. The double‐treatment arm showed the highest degree of necrosis, indicating better response (average 20% necrosis in primary tumors), followed by sunitinib (14%), everolimus (7.5%) and then MK571 and untreated groups (2–5%). Upon comparing the distant organ metastasis among the different groups, the sunitinib + MK571 and everolimus groups showed the lowest level of metastasis among the treated mice (25–30%) (number of mice showing distant organ metastasis in their group). Even though the everolimus group showed a low level of lung metastasis, it was less tolerated by mice as 50% (2/4) were deceased halfway through the treatment period. The sunitinib and MK571 groups showed 50% and 75% metastasis, respectively (Table S1).

### *In vivo* validation, blocking ABCC2 increases sunitinib uptake in CAKI‐2 tumor cells

**Figure 6:**
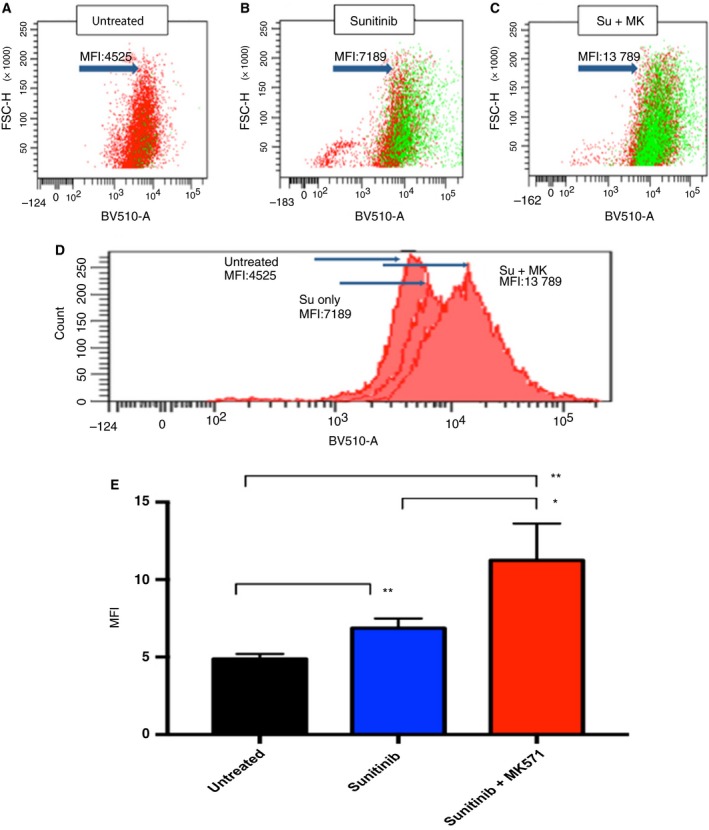
(A) *In vivo*, flow cytometry shows sunitinib uptake is increased with blockage of ABCC2, (A–C). Dot plot showing median fluorescence intensity of intracellular sunitinib in different groups. (D) Histogram showing fluorescence intensity shift with dual treatment. (E) Bar chart showing significant increases in sunitinib uptake after ABCC2 blockage in the double‐treatment mice group (Untreated *n* = 3, sunitinib *n* = 3, sunitinib + Mk571 *n* = 4). Two‐tailed t‐test used for the analysis, error bars represent SD (nonsignificant: ns, *P *>* *0.05:*, *P ≤ *0.05:**, *P *≤* *0.01:***, P ≤* *0.001:****, *P *≤* *0.0001).

Tumors grown in mice corresponding to the different treatment groups were harvested at the experiment end point (after 8 weeks of treatment). After tumor dissociation into a single‐cell suspension, the sunitinib content of each mouse tumor was measured by flow cytometry as described in previous sections. Our *in vivo* analysis yielded similar results to what was detected with *in vitro* treatment, with a significant increase in intracellular sunitinib among the group that was additionally treated with the ABCC2 blocker. sunitinib median fluorescent intensity (MFI) was 11 250 in the sunitinib + MK571 treated mice versus 6870 in the sunitinib only treated mice (*P* = 0.028) (Fig. 6).

## Discussion

In the era of personalized medicine, we are getting to understand more about the individualized biology of different tumors and our approach to this management should reflect this knowledge. While there are many drugs under development toward that goal, we are not yet there for the different RCC subtypes (Posadas *et al*., 2017). Currently, there are no evidence‐based guidelines for appropriate management of nonclear cell RCC (Ciccarese *et al*., 2017; Giles *et al*., 2017). The most common of these nonclear cell RCC subtypes is PRCC, which of itself is known to harbor at least two histological types PRCC1 and PRCC2 with different molecular makeup and clinical behavior (Saleeb *et al*., 2016; TCGA, 2016; Yang *et al*., 2005). There is an unmet need to treat these tumors in a manner relevant to their biology so as to respond better to therapy.

There are ongoing clinical trials on the use of MET inhibitors on PRCCs that harbor an activation in the MET pathway (Giles *et al*., 2017). While PRCC1 is known to harbor MET overexpression, PRCC2 is reported to have activations in the NRF2‐ARE pathway (Ooi *et al*., 2013; Saleeb *et al*., 2016; TCGA, 2016). Albiges *et al*. reported some increased MET activity in PRCC2 in comparison with the normal kidney and CCRCC. Nonetheless, they failed to find MET amplification or a considerably higher MET expression by (q‐RT‐PCR) in PRCC2 (Albiges *et al*., 2014). The MET pathway was not one of the PRCC2‐enriched pathways, established by the TCGA and ourselves in studying the KIRP cohort (TCGA PRCC cohort) (Saleeb *et al*., 2016; TCGA, 2016). There are currently no particular clinical trials targeting the more aggressive PRCC2 tumors.

We have previously studied the PRCC subtypes and found pathways enriched in each type that can be relevant for therapy. In the current study, we focus on the more aggressive PRCC2 where we have detected a high level of ABC drug transporters (predominantly ABCC2) and enrichment in the mTOR pathway, consistent with TCGA findings (Saleeb *et al*., 2016; TCGA, 2016), as ABCC2 is known to contribute to drug resistance (also called multidrug‐resistant protein (MRP2). We hypothesized that blocking ABCC2 would enhance the cancer response to the current first‐line therapy (VEGF TKI, e.g., sunitinib) (Tivnan *et al*., 2015). We also assessed the validity of using mTOR inhibitors (current second‐line treatment) in PRCC2. We identified an RCC cell line that phenotypically and molecularly closely represents PRCC2. We compared the CCLE gene expression data of 20 RCC cell lines to PRCC2 transcriptomic signatures. Our analysis highlighted the CAKI‐2 cell line as best match for PRCC2 regarding the expression of the top expressed genes as well as the expression of the ARE pathway genes. We confirmed these results by analyzing the *in vivo* CAKI‐2 cell morphology and immunoprofile which matched those of PRCC2 that was also consistent with literature (Brodaczewska *et al*., 2016). CAKI‐2 is not known to harbor VHL mutations or 3P deletions distinguishing it from CCRCC. Brodaczewska *et al*. described multiple evidence linking CAKI‐2 to PRCC (Brodaczewska *et al*., 2016). Additionally, CAKI‐2 has been known to harbor chromosome 8 aberrations, also described in PRCC2 (Furge *et al*., 2007; Saleeb *et al*., 2017).

As expected, CAKI‐2 responded significantly better to anti‐VEGF TKI sunitinib treatment after blocking ABCC2 *in vitro*. Similarly, the double‐treatment arm showed the best response *in vivo*, with the smallest average tumor size, highest percentage of apoptotic cells, lowest proliferation rate, highest rate of tumor necrosis, and low tumor metastasis rate. We confirmed that this effect is related to an increase in sunitinib uptake, which we have demonstrated both *in vitro* and *in vivo*. Contrarily ABCC2‐low RCC cell lines (CAL‐54) did not show an increase in sunitinib uptake upon ABCC2 blockage. The results ascertain what was shown in other studies regarding an increase in drug uptake upon ABC transporter blockage, especially in cancer with a high level of transporters (S. Shukla *et al*., 2011).

A known obstacle in the treatment of TKIs is the development of resistance. One of the proposed mechanisms for resistance is their active cellular efflux induced by the ABC transporters (He and Wei, 2012). Many TKIs are substrates for ABC transporters, which produces a complex intricate relationship between TKIs and ABC transporters. ABC transporters would contribute to TKI resistance while TKIs could reversibly inhibit the ABC transporter efflux mechanism (He and Wei, 2012). There are early trials assessing individualized sunitinib dosing in RCC, which have shown improved PFS with the increase of sunitinib dosage when tolerated (Kumar and Kapoor, 2017). This could be possibly linked to blocking the ABC transporter resistance by the increase in TKI exposure (Kathawala *et al*., 2015; Suneet Shukla *et al*., 2009). Conversely, an addition of an ABC transporter blocker could enhance the therapeutic potential of TKIs(S. Shukla *et al*., 2011; Suneet Shukla *et al*., 2009).

Interestingly, ABCC2 blockage alone by MK571 also affected the PRCC2 cell line growth both *in vitro* and *in vivo*, in keeping with emerging evidence suggesting that ABC transporters contribute to tumor growth in ways beyond drug efflux (Fletcher *et al*., 2010; Nozaki *et al*., 2010). Henderson *et al*. demonstrated that in neuroblastoma, ABCC transporters contribute to the tumors biology and clinical behavior independent of its role as chemotherapy resistance (Henderson *et al*., 2011). It is proposed that ABC transporters contribute to a number of hallmarks essential for cancer initiation and progression, as proliferation and apoptosis, cell differentiation and stem cell maintenance, cell migration invasion and metastasis (Fletcher *et al*., 2010). Montelukast is an FDA‐approved leukotriene receptor antagonist that functions similarly to MK571 and is found to also inhibit ABCC2 (Roy *et al*., 2009). Montelukast was found to have a chemotherapeutic potential in a number of studies, highlighting the promising role of ABCC transporter blockers as cancer therapeutic agents(Tsai *et al*., 2017).

Likewise, we assessed the effect of the mTOR inhibitor everolimus on the PRCC2 (CAKI‐2) RCC cell line. *In vivo*, the everolimus‐treated arm showed the second best response to therapy after the sunitinib+ blocker group. Everolimus is currently among the second‐line therapies of clear cell RCC, a paradigm that is also generally adopted for the nonclear cell subtypes. Our data indicate that in pure PRCC2 biology, everolimus does show better chemopreventative effect than sunitinib. Two previous trials (ASPEN and ESPN) have attempted to compare between sunitinib and everolimus treatment in PRCCs (Armstrong *et al*., 2016; Schmid and Gore, 2016; Tannir *et al*., 2016). In both the ASPN and ESPN trials, no attempt was made to subtype the PRCC patients or distinguish between the treatment arms in the specific PRCC subtypes. Additionally, the number of PRCC patients in each of these trials was considerably small with 70 patients in the ASPEN trial (33 sunitinib and 37 everolimus) and 27 in the ESPN trial (14 sunitinib and 13 everolimus). Ravaud *et al*. in the SUPAP trial assessing the effect of sunitinib treatment only on the two PRCC subtypes found slightly superior benefit with type 1 versus the type 2 (better partial response, longer stable disease, and overall survival). These data highlight potential benefit in subtyping PRCCs in clinical trials. Large subtype‐specific clinical trials are required to formulate better guidelines (Giles *et al*., 2017).

## Conclusion

We believe our study uncovers extremely pertinent information regarding PRCC2 treatment. Discoveries on the individualized nature of tumor biology should direct the clinical field toward similarly individualized treatments. We assessed the efficacy of targeting tumor‐specific pathways in a preclinical setting and showed that these treatment modalities show great promise. We highlight the importance of targeting ABC transporters, as ABCC2 in PRCC2 and in other high drug transporter tumors. We acknowledge that the current study has a number of limitations, particularly its experimental design in cell lines and the nature of the assessment in *in vitro* setting and in mouse models. However, our findings would pave the way for clinical trials that can confirm these results and lead to a more accurate tumor‐specific treatment for the patients of PRCC2 and similar tumors.

## Author contributions

RS designed the project, performed the experiments, analyzed the data, and wrote the manuscript. MF performed *in vivo* and *in vitro* experiments and analyzed data. ZL guided and performed experiments, and interpreted results. FB reviewed and helped interpret the tumor pathology data. JB helped with the *in vivo* experiments. GB reviewed data and guided the tumor therapeutic experiments, and helped interpret data. AF reviewed and helped interpret the clinical data. FB performed experiments and immunohistochemical stains. MD: reviewed and helped interpret the tumor pathology data. GS, senior scientist, supervised the project, guided the experimental framework, helped interpret the results, and reviewed the final work and the manuscript.

## Supporting information


**Fig. S1.** The CAL‐54 RCC cell line represents PRCC1.
**Fig. S2.** The CAKI‐2 cell line represents PRCC2.
**Table S1.** Histological examination of mice tumor models.
