# Supplementary material for: Modulating ATP binding cassette transporters in papillary renal cell carcinoma type 2 enhances its response to targeted molecular therapy
Source: Mol Oncol. 2018 Aug 23;12(10):1673–88. doi: 10.1002/1878-0261.12346 (PMC6165997; doi:10.1002/1878-0261.12346)
Supplement: Supplementary file 1 — Fig. S1. The CAL‐54 RCC cell line represents PRCC1. Fig. S2. The CAKI‐2 cell line represents PRCC2. Table S1. Histological examination of mice tumor models. [file MOL2-12-1673-s001.pdf]

Supplementary Figure 1:

A. **B.**

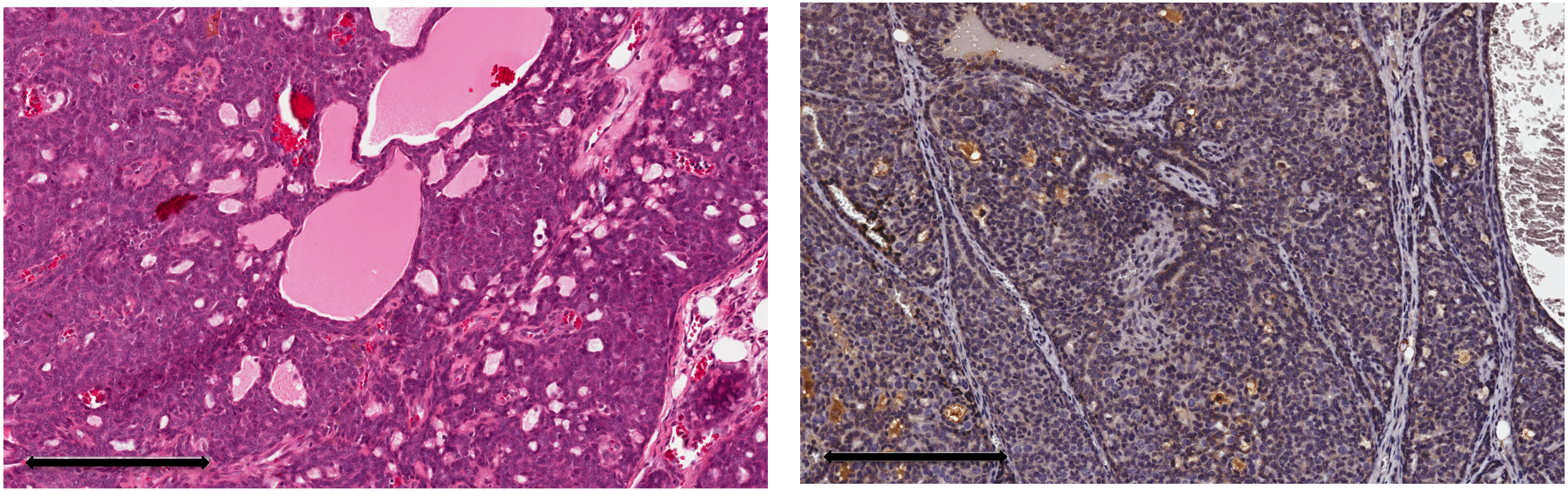

Supplementary Figure 1. The CAL-54 RCC cell line represents PRCC1. A) Histologic examination of sections of these cells xenografted in mice shows tubulo-papillary with small cells, scant cytoplasm and inconspicuous nucleoli consistent with PRCC1 morphology, B). The cells stained negative for ABCC2 IHC also consistent with PRCC1 tumors. Scale bar = 200 um

Supplemental Figure 2:

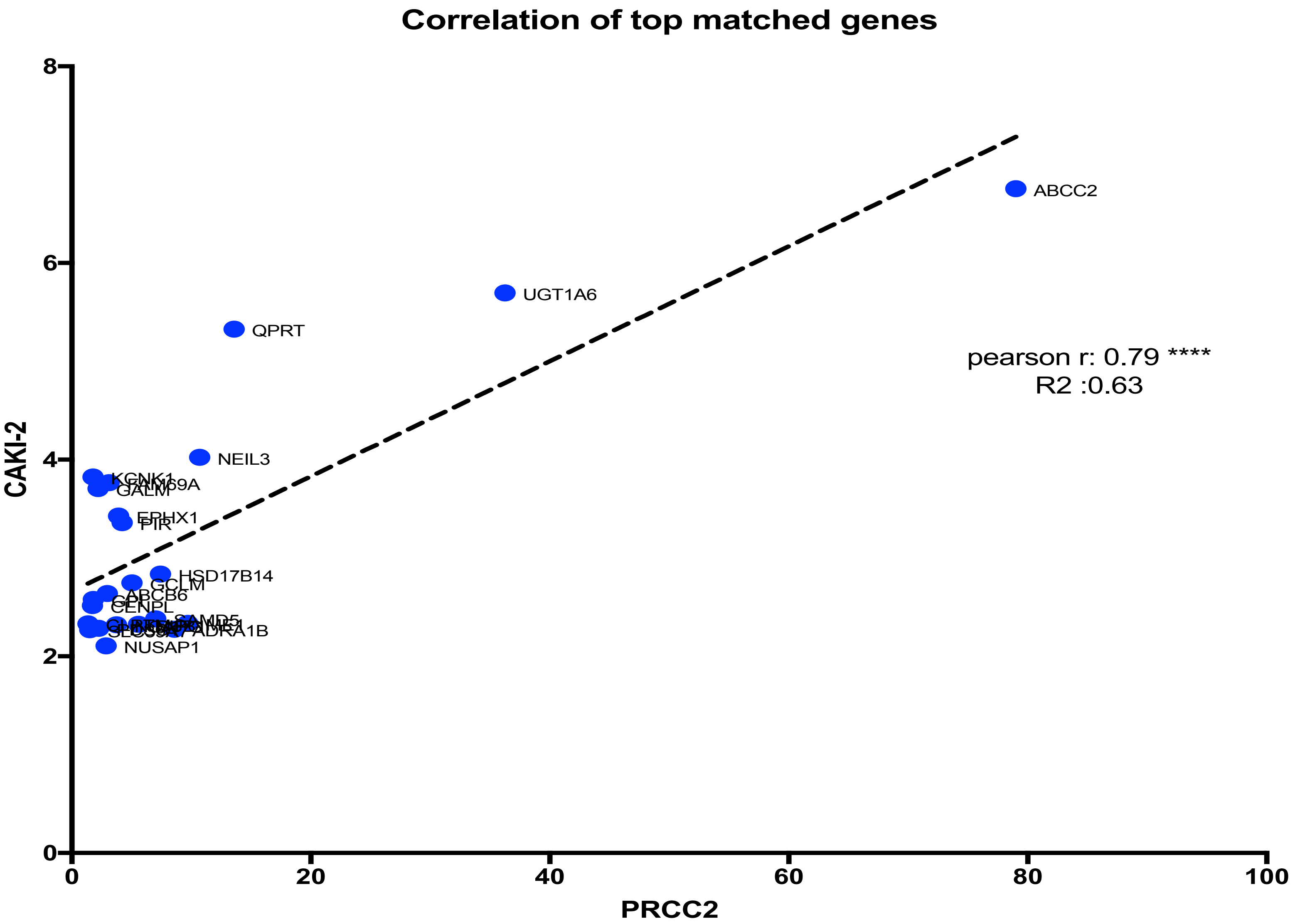

Supplementary Figure 2. The CAKI-2 cell line represents PRCC2. CAKI-2 shows highly significant positive correlation with PRCC2 on top differentially expressed genes.

Supplementary Table 1: Histological examination of mice tumor models

Treatment groups	Histological pattern	Average Necrosis	Local invasion	Metastasis (Lung)	Lung	Kidney	Liver
Untreated	Papillary architecture	2-5%	All locally invasive	25-30%	Mild to moderate inflammation	No Inflammation or necrosis	
MK571		2-5%		75%			
Sunitinib		14%		50%			
Everolimus		7.5%	75% locally invasive	25-30%	Severe acute inflammation in 50% of mice		
Sunitinib + MK571		20%	63% locally invasive	25-30%	Mild to moderate inflammation		
